# Concurrent pulmonary TB and histoplasmosis in an HIV-negative patient with Evans Syndrome

**DOI:** 10.5588/ijtldopen.24.0607

**Published:** 2025-03-12

**Authors:** T.-W. Kao, M.-T. Tsai, S.-C. Pan, C.-C. Shu, W.-Y. Liao, J.-S. Jerng

**Affiliations:** Department of Internal Medicine National Taiwan University Hospital and National Taiwan University College of Medicine, Taipei, Taiwan.

**Keywords:** tuberculosis, autoimmunity, *Histoplasma capsulatum*, haematological complications

Dear Editor,

We report on a 74-year-old male who presented with a 2-month history of fever, productive cough with only whitish but not blood-tinged or yellow sputum, and weight loss exceeding 5 kg (about 8% of original body weight) in the past 3 months. He had previously worked as a cement labourer for decades and had only an underlying disease of hypertension. He was a never smoker. Initially, the patient presented to a regional medical institution in Northern Taiwan and was later referred to our tertiary centre for a workup. Upon examination, he was afebrile under antipyretics and haemodynamically stable with intact saturation on pulse oximetry but appeared to be cachectic and have mild jaundice. A detailed examination revealed coarse crackles over the left lung field, which were otherwise non-contributory. Initial haemogram documented normocytic anaemia with haemoglobin at 6.5 g/dL and mean corpuscular volume at 91.8 fL, and biochemistry showed indirect hyperbilirubinemia (total bilirubin at 1.63 mg/dL; direct type 27.0%), hyponatremia at 129 mmol/L, and hypokalemia at 4.1 mmol/L. A chest X-ray revealed an oval mass shadow over the left peripheral lung field and a small consolidation around the aortic arch **(**Figure Panel A**)**, and computed tomography showed a 2.2 cm lobulated, solid subpleural nodule in the left lower lobe and small para-aortic consolidations, along with paratracheal lymphadenopathies (Figure Panel B+C). Sputum culture yielded Gram-negative bacilli and yeast-like organisms.

Given the possibility of a lung tumour, the patient was admitted for further diagnosis. Endobronchial ultrasound-guided transbronchial needle aspiration was performed at the group 4R heterogenous mediastinal lymph node, and positive polymerase chain reaction (Xpert MTB/RIF Ultra assay) revealed *M. tuberculosis* complex without rifampicin resistance. Tissue culture of the mediastinal sample showed *Streptococcus parasanguinis* and *Candida albicans* enrichment. The pathology of formalin-fixed specimens revealed anthracosis and histiocytes without atypical cells. Further real-time ultrasound-guided biopsy was performed over another level IV cervical lymphadenopathy with heterogeneous echogenicity and the 2 cm hypoechoic lung nodule. Caseating granulomatous inflammation was detected microscopically in the cervical lesion. Granulomatous inflammation was evident in the lung tumour **(**Figure 1D**)**, but only a few acid-fast bacilli were present (Figure Panel E). In addition, intracellular budding yeasts were highlighted in the lung nodule specimen using periodic acid–Schiff reagent (Figure Panel F) and Gomori’ methenamine-silver stain (Figure Panel G), but mucicarmine and Fontana–Masson stain were negative. Tissue cultures were negative, and acid-fast staining and mycobacterium culture collected from sputum, cervical and mediastinal lymphadenopathies, as well as peripheral pulmonary lesion, all rendered negative, whereas tissue culture of the lung nodule yielded *Histoplasma capsulatum*. The patient was eventually diagnosed with concurrent pulmonary TB and histoplasmosis despite the denial of any travel or contact history. HIV screening was negative, and the immunoglobulin level showed no deficiency. Standard anti-TB therapy with rifampicin, isoniazid, pyrazinamide and ethambutol was initiated for TB. Within 2 days day of initiating anti-TB medications, rifampicin was replaced with levofloxacin because of hyperbilirubinemia and drug–drug interaction with itraconazole, which was added on the ninth day to treat the histoplasmosis. Intriguingly, unconjugated hyperbilirubinemia prompted an additional workup, revealing a low reticulocyte production index, low haptoglobin, and positive Coombs’ test with anti-Wra auto-antibody, indicating autoimmune haemolytic anaemia. In addition, anti-glycoprotein Ib/IX was positive, and thrombocytopenia progressed, consistent with the diagnosis of idiopathic thrombocytopenic purpura (ITP). The co-existence of autoimmune haemolytic anaemia and ITP led to the diagnosis of Evans syndrome. Isoniazid was discontinued because his hemolytic anaemia was aggravated despite the introduction of azathioprine. After additional administration of dexamethasone 0.8 mg/kg/d 5 days later, his haemogram eventually stabilised and subsequently normalised under azathioprine 100 mg/d and prednisolone 10 mg/d. Isoniazid was rechallenged one month after discharge, and a final anti-TB regimen was composed of additional fluoroquinolone, pyrazinamide, and ethambutol for an expected 12–18-month treatment (Table). Itraconazole is expected to complete at least a 12-month course. The patient is currently free from symptoms, and haemogram, haptoglobin and bilirubin levels all returned to normal.

**Figure.**
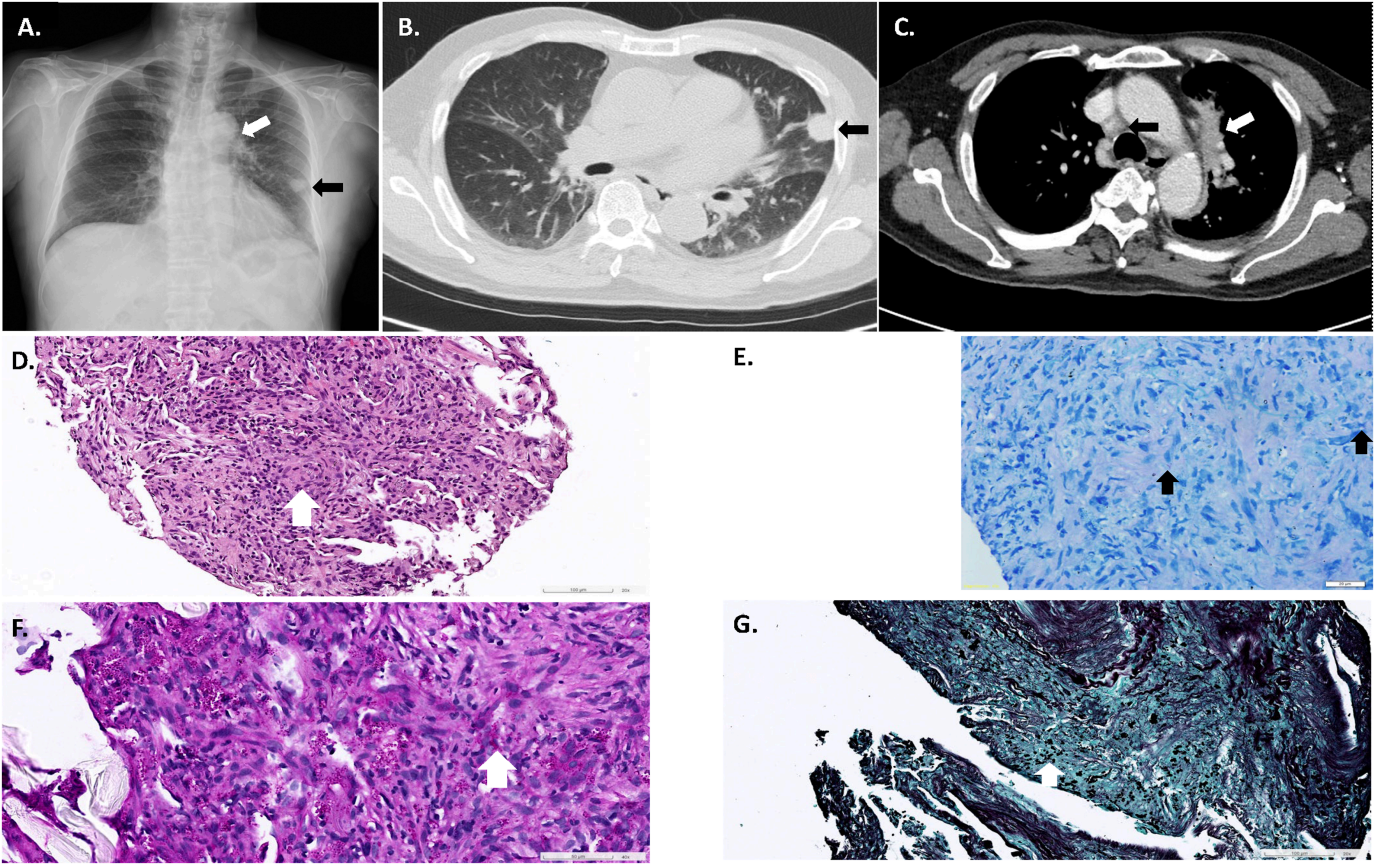
Case images **A)** Chest plain film (black arrow: peripheral lung lesion; white arrow: para-aortic consolidation). **B)** Chest computed tomography, axial view, in lung window (black arrow: peripheral lung lesion) and **C)** mediastinal window with contrast enhancement (black arrow: mediastinal group 4R lymph node; white arrow: para-aortic consolidation). **D)** Hematoxylin and eosin stain showed granulomatous inflammation (white arrow). Amplification: 20x. **E)** Few bacilli (black arrow) identified by acid-fast stain (amplification 40x). **F)** Positive stain of yeast (white arrow as an example) in Periodic acid–Schiff stain (amplification: 40x). **G)** Positive stain of yeast (white arrow as an example) by Gomori’ methenamine-silver stain (amplification: 20x).

**Table.** Timeline of diagnosis and treatments in days for TB, histoplasmosis and Evans syndrome.

Diagnosis	D0 (TB)	D1	D2 (Evans)	D3	D4	D5	D6	D7	D8	D9 (histoplasmosis)
Anti-TB	H, E, R, Z		E, Z, Q							
Anti-histoplasmosis										-azole
Anti-Evans			Azathioprine					Azathioprine+dexamethasone		

H = isoniazid; E = ethambutol; R = rifampin; Z = pyrazinamide.

Both TB and histoplasmosis are under-recognised yet potentially life-threatening infections.^1^ Concurrent infection with both pathogens, which we believe has not been reported before in an immunocompetent host, presents significant challenges in diagnosis and treatment. The clinical presentations and imaging characteristics of these infections were notably similar, leading to challenging differential diagnoses. We comprehensively reviewed the literature on cases with concomitant TB and histoplasmosis in patients with immunodeficiency, predominantly in the presence of HIV infection. Two case reports documented such co-infection in an adult^2^ and an adolescent,^3^ but no haematological consequences were present. To our knowledge, this is the first reported case of concomitant TB and histoplasmosis potentially complicated with Evans syndrome in an HIV-negative patient. Co-infection might contribute to divergent clinical trajectories and pulmonary presentations. In this case, both respiratory symptoms and fever improved under anti-TB treatment but disappeared until the initiation of itraconazole, indicating that the systemic symptoms could be attributed to both *Mycobacterium tuberculosis* and *Histoplasma*. Positive culture of *Histoplasma* further implied the pronounced burden of fungal infection. Soil has been proposed as the natural reservoir for *Histoplasma* spp., with previous outbreaks thought to be associated with soil disruption.^4^ However, there is a growing recognition of cases among individuals outside endemic regions, facilitated by increased awareness and enhanced diagnostic capabilities. There is a lack of comprehensive epidemiological data on global and regional prevalence rates. In this case, given the patient’s lack of foreign travel or exposure to known cases, the most likely source of infection may be linked to his occupation as a cement worker, which involved frequent soil contact. TB remains relatively common in Taiwan,^5^ particularly among the elderly, and the source of infection is often unidentified.

The concurrent occurrence of anaemia and thrombocytopenia in patients with TB or histoplasmosis is common, but the causal relationship between these conditions remains poorly understood. Albeit rare, a previous study has documented secondary autoimmune haemolytic anaemia caused by TB disease, characterised by dysregulated auto-antibodies targeting red blood cells.^6^ In a previous study conducted in endemic areas, positive auto-antibody titers were present in 32% of individuals with TB.^7^ In our case, haematological alterations emerged following pathology-confirmed diagnoses of TB and histoplasmosis but prior to the initiation of antimicrobial therapy. Haematological parameters were restored only after the administration of immunosuppressants and the achievement of effective infection control, suggesting that these infections may contribute to acute haematological compromise. Management of Evans syndrome in such cases is challenging, as the therapeutic regimen requires adjustments. The treatment regimen must be carefully selected to minimise compromising therapeutic efficacy while avoiding the induction or worsening of haematological complications. Six independent cases of concurrent TB and Evans syndrome have been previously reported,^8^ but none involved additional fungal infections. Both the underlying mechanism leading to a potential causal relationship and the long-term clinical outcomes for such patients remain unknown, highlighting the need for continuous follow-up of both pulmonary and haematological status.

In conclusion, concurrent TB and histoplasmosis in this non-HIV patient caused serious pulmonary infection and haematological complications, like Evans syndrome, as exemplified by the clinical diagnosis and treatment challenge. Such possibilities should be considered when managing concurrent pulmonary infection and haematological abnormality in an immunocompetent patient. In addition, the causal relationships between TB, histoplasmosis and Evans syndrome require further study.
